# *Ugni molinae* Fruit as a Source of Bioactive Compounds with Good Quality Traits

**DOI:** 10.1155/2021/6683877

**Published:** 2021-04-23

**Authors:** Ricardo I. Castro, Patricio Ramos, Carolina Parra-Palma, Luis Morales-Quintana

**Affiliations:** ^1^Multidisciplinary Agroindustry Research Laboratory, Instituto Ciencias Químicas Aplicadas, Facultad de Ingeniería, Universidad Autónoma de Chile, Talca, Chile; ^2^Instituto de Ciencias Biológicas, Universidad de Talca, Talca, Chile; ^3^Núcleo Científico Multidisciplinario-DI, Universidad de Talca, Talca, Chile; ^4^Programa de Doctorado en Ciencias Mención Ingeniería Genética Vegetal, Universidad de Talca, Talca, Chile; ^5^Multidisciplinary Agroindustry Research Laboratory, Instituto de Ciencias Biomédicas, Facultad de Ciencias de la Salud, Universidad Autónoma de Chile, Talca, Chile

## Abstract

Since the intake of fruits and vegetables displays important effects on the incidence of several chronic diseases in humans, consumers' attention worldwide is focused on the identification of functional foods. In this sense, *Ugni molinae* (murtilla or murta fruit) is an important source of molecules with a strong antioxidant capacity that is widely used as a medicinal plant in Southern Argentina-Chile. Research on murtilla berries showed that this fruit and its leaves can be an excellent source of polyphenols and bioactive compounds with antibacterial and antioxidant capacity. This review is aimed at providing valuable information and discussing the available literature focused on four principal points: (i) fruit quality and plant physiology, (ii) compound content with bioactive properties, (iii) health properties for consumers of the fruit and leaves, and (iv) challenges for future research. Based on these four points, we propose that murtilla fruit can be a potential ingredient for new functional food products.

## 1. Introduction

Murtilla, murta, or Chilean guava are the names given to the *Ugni molinae* Turcz plant. In the Mapuche language of Southern-Chile native inhabitants [1–3], it is called by the name of Uñi, which is a native Chilean and Argentinean species belonging to the Myrthaceae family. Murtilla is a new berry that has the potential not only to become a fruit that Chile and/or Argentina can export but also to become a functional food. Murta is a wild shrub growing in the central-southern region of Chile, especially in the Coast Mountain and the pre-Andean mountains between the Maule and Aysén regions, including the Archipelago of Juan Fernández. In Argentina, it grows in the Patagonian region [2, 4].

Murtilla is consumed as a fresh fruit because of its organoleptic characteristics, although it is also processed by the food industry for making juice, canned items, confections, jams, and liquor products [3]. This fruit has potential beneficial effects on human health and may be used as a preservative in food products [3, 4]. In this line, murtilla berries showed a higher content of the total beta-carotene, phenolic compounds, and flavonoids [5]. These compounds confer a high antioxidant capacity. For example, as evaluated by ORAC, the antioxidant capacity was observed in fresh fruit; however, when the fruits were treated with high temperatures (70–80°C), the antioxidant properties were maintained, indicating a high thermal stability [5]. Additionally, another example of a health benefit of murtilla fruits is its beneficial effects on the management of cardiovascular disease as described by Singleton et al. in 1999 [6]. With respect to the leaves, chemical components with antioxidant and anti-inflammatory capacity have been reported, indicating that the fruit can be used for health benefits [7–9]. For this reason, the present review is aimed at critically summarizing information of the past and recent years on the characteristics of the shrub, its fruit quality, physiology, and beneficial effects on health.

## 2. Plant and Fruit Morphology and Growth

**Figure 1:**
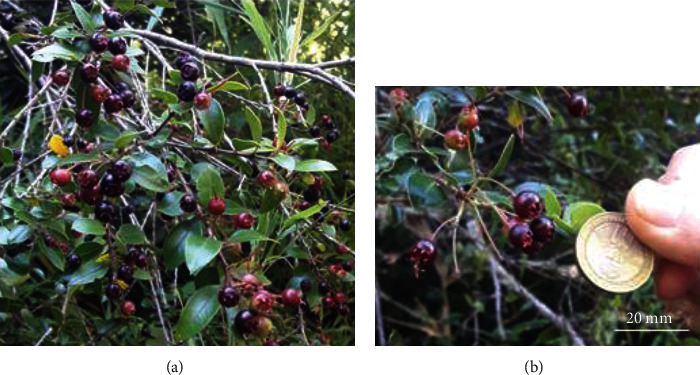
Murtilla fruit. (a) Murtilla fruit in different developmental stages visible from the same plant. (b) Color and relative size of the murtilla fruit in ripe stage. Picture was taken in the mountains of Choshuenco (39°50′11^″^S–72°4′57^″^W), Los Rios region, Chile.

The plant of murtilla is a polymorphic shrub of great foliage. The murtilla shrub can grow to approximately 2 meters. Its branches are compressed, covered by hairs, and have ascending stems and branching [10]. Its leaves are between 2 and 2.5 cm in length. The form of the leaf is petiolate, opposed, without stipules, ovate-oblong, with a sharp apex, green lamina, glaucous, and has marks on the underside [10]. Its pendulous flowers are hermaphroditic, axillary, solitary, and long pedunculated [10]. There are five sepals united at the base and bent outwards; five linear petals, sharp and rounded; numerous stamens; and a style longer than these. The flowering season is between November and December, while the fruit production is between February and April [10]. The murtilla plant is an evergreen plant that produces a small globoid berry fruit with an equatorial diameter of 7–13 mm. The unit weight reaches 0.45 g [10]. Its pulp is white, and depending on the different plant ecotypes, it develops a variety of fruit colors including soft green, yellow, fuchsia (purplish), and light and dark red [3, 10–12] (Figure 1).

## 3. Fruit Quality

The murtilla plant may grow in the wild or may be cultivated. Thus, murtilla fruits are consumed fresh due to its organoleptic characteristics, and they are also used for the preparation of jams, syrups, desserts, and liquors [10]. With respect to the quality of a fruit, it is defined as the set of internal and external features inherent to the fruit, thereby determining consumer acceptability. These characteristics, which are also known as “quality criteria” for the consumer, include appearance (size, color, and shape), texture, nutritional value, safety [14], flavor, and aroma [15, 16], and these characteristics of the murtilla fruits have been partially studied, defining some of those quality properties.

### 3.1. Aroma

The aroma is strongly related with the fruit flavor and is a critical component of the perceived fruit quality [16–18]. The aroma of fruits is determined by a large number of volatile compounds of different molecular types (alcohols, terpenes, esters, aldehydes, and others) that are part of the flavor; additionally, its biosynthesis involves different pathways, and depends on many factors, such as ripening stage, cultivar, agronomic management, and harvest and postharvest conditions [17, 19].

With respect to the aroma of murtilla fruits, the ripe murtilla fruit exhibits a special and surrounding aroma, although the volatile compounds have not yet been identified completely. However, the first description of the aroma compounds of murtilla fruits was provided by Scheuermann et al. in 2008. The authors showed that the volatile compounds identified in murtilla fruit aroma have been found in other aromatic fruits (tropical or traditional fruits) widely consumed around the world. To obtain the identification of the volatile compounds from murtilla fruit, the authors obtained fruit from 2.5-year-old murtilla plants from four ecotypes. These ecotypes were selected based on their agronomical and organoleptic characteristics. The major concentrations of volatile compounds in murtilla fruit aroma produced by the four ecotypes are as follows: methyl 2-methyl butanoate, ethyl butanoate, ethyl 2-methyl butanoate, methyl hexanoate, ethyl hexanoate, methyl benzoate, and ethyl benzoate characteristics (Table 1). Out of these compounds, the authors showed that the most important compounds are the ester types independent of the ecotype studied and similar to those of other berries described previously as strawberry or blueberry.

### 3.2. Flavor, SSC, TA, Postharvest Life, and Nutritional Properties

For consumers, one of the most important determinants of the fruit quality is the fruit nutritional composition. In this sense, the sugar contents are determined from the fruit quality. During fruit development and ripening, the fruits can accumulate sucrose, fructose, and glucose compounds [20]. In 2009, González Enei [21] evaluated the soluble solid content (SSC) from murtillas fruit obtained from different growing areas of the Maule region in the center of Chile. The author observed considerable differences in the SSC values, with differences between 10 and 27 °Bx. Even when the differences were considerable, these are still very high SSC values, indicating the considerable sweetness of the fruit. Additionally, murtilla fruit contains a high amount of dietary fiber (near to 20.0%) [22] (Table 2). In several fleshy fruits, the SSC and titratable acidity (TA) are two important properties related with ripening, and they are the main determinants for fruit flavor [4, 16, 23]. Along this line, Table 2 shows SSC values of 6.5-28 °Bx and TA values of 3.5–5.2 pH.

Other important components in berries are the vitamin and mineral concentrations. Unfortunately, there are only a few reports that are related to the vitamin and mineral contents of murtilla fruit or leaves. Along this line, in 2008, Moraga [24] described that murtilla berries contain around 4.53 to 12.55 mg of vitamin C in 100 g of fresh berries. These values are low compared to other fruits such as blueberry. With respect to the mineral content, Merino in 2002 [25] and previously Schmidt-Hebbel et al. in 1990 [26] described that murtilla ripe fruit can accumulate 90 mg of calcium, 20 mg of phosphorus, and 116 mg of the potassium minerals (Table 2). A high water content of around 77 to 85% was documented in murtilla fruit, together with other nutritional components as shown in Table 2.

With respect to the postharvest life, a comparative study of the two most important commercial murtilla varieties (namely, South Pearl INIA and Red Pearl INIA) showed that South Pearl INIA has a short shelf life of 35 days of storage at 0°C [4]. In 2019, Fuentes et al. [4] reported that in a postharvest life assay for this two Murtilla varieties, Red Pearl INIA has a major potential for postharvesting after 20 days at 0°C when compared to the South Pearl INIA variety.

### 3.3. Principal Bioactive Compounds Described from Murtilla Fruit

Polyphenolic compounds (PCs) are important not only in terms of quality but also in terms of the PCs influence over visual fruit appearance related with fruit or vegetable color, and their influence over the taste [4, 16, 27, 28]. Additionally, from a biomedical point of view, the PCs have gained a great interest during the last decade because they appear to be associated with the prevention of different diseases [29].

The main PCs reported in murtilla fruits and leaves are shown in Table 3. Highlighting the most important PCs found in different plant parts, we have caffeic acid 3-glucoside, ferulic acid, kaempferol, kaempferol-3-glucoside, gallic acid, myricetin, and others, such as rutin, quercitrin, luteolin, luteolin-3-glucoside, and p-coumaric acid (Table 3). Different reports showed that the murtilla fruit has 32 *μ*mol GAE g^−1^ FW of total PCs, using the Folin-Ciocalteu method, while other reports showed that it has 34.9 mg GAE g^−1^ DW of total PCs [30], these values being considered a high concentration compared to the commercial blueberry with 17 *μ*mol GAE g^−1^ FW [31]. However, in another study, the authors showed that the murtilla fruit has only 9.2 mg GAE g^−1^ DW (Table 3). Along this line, in 2011, Arancibia-Avila et al. [32] showed that the levels of PCs of murtilla fruit are significantly higher than the most popular berries such as blueberries or raspberries. The antioxidant activities in murtilla were high in methanol extract when compared to the other berries, and the authors showed that the highest ability to scavenge free DPPH radical was found for murtilla nonripe methanolic extract, with values of 31.55 ± 1.4 mg GAE/g for polyphenols and 5.22 ± 0.3, 12.16 ± 0.6, and 2.24 ± 0.1 mg CE/g for flavonoids, tannins, and flavanols, respectively [32].

#### 3.3.1. Anthocyanins Found in Murtilla Fruit

**Figure 2:**
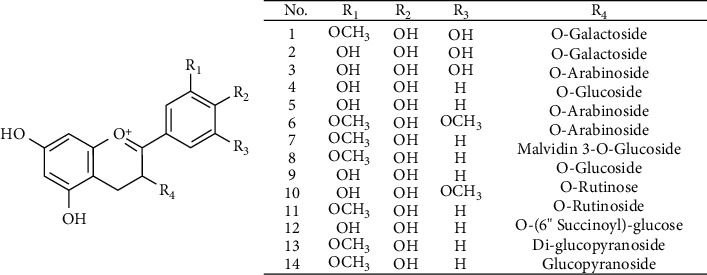
Structures of identified anthocyanins from murtilla fruit. The number of each structure is the same number given to the corresponding anthocyanin molecule described in Table 4.

Anthocyanins are naturally present in vegetables and fruits, and one of the most abundant types of these PCs are named flavonoids [29]. These compounds are vegetal pigments that promote health benefits to consumers due to their high antioxidant capacity [29, 33], and they are responsible for the blue, purple, and red colors in the flowers, vegetables, and fruits [27, 29]. To date, more than 600 anthocyanins have been identified in nature [29], including the anthocyanins described in murtilla fruit as shown in Table 4 along with their corresponding structures shown in Figure 2.

Recently, in 2014, Brito et al. [34] evaluated the anthocyanin content in three different Chilean native berries (calafate, murtilla, and arrayán fruits). The authors reported that murtilla berries had a smaller anthocyanin content when compared to arrayán or calafate fruits with values of 6.9, 15.2, and 51.6 (mg cyanidin 3-O-glucoside g^−1^ DW), respectively, even though a higher flavonoid content has been described in the Myrtaceae family [30, 31] (including murtilla fruits). Now, with respect to the principal anthocyanins found in murtilla ripe fruit, after an extraction in methanol-HCl and/or methanol, it was possible to identify malvidin-3-, delphinidin-3-, and peonidin-3-arabinoside; peonidin-3- and malvidin-3-glucoside (Table 4) [35].

## 4. Benefits to Consumer's Health

The PCs, and in particular the anthocyanin compounds, are effective antioxidant compounds with different beneficial health effects, such as antiallergic, antimicrobial, anti-inflammatory, antiplatelet, anticarcinogenic, antimutagenic, and neuroprotective effects [4, 29, 36]. For this reason, multiple studies have been carried out searching for high levels of anthocyanins in different fruits. The berry fruits are interesting targets, because of their high concentrations of PCs and anthocyanins. Of course, this is the same for murtilla fruit due to its high concentrations of PCs. However, it is necessary to understand that the richness of any PCs found in each fruit or food is not necessarily related with the correct absorption in the human organism; for these reasons, the bioavailability of each PC should be studied to correlate the intake and the effects thereof [29]. In this sense, in 2018, Ah-Hen et al. [37] showed that at the end of the small intestine digestive step, the bioaccessibility index of PCs found in fresh murtilla berries and murtilla fresh juice achieved a relatively high value (around 70%). More specifically, the authors showed that the fresh murtilla fruit increased the release of bioactive compounds in the small intestine, while the murtilla fresh juice released the bioaccessible bioactive compounds in the gastric stage [37]. Between the different beneficial properties described, the murtilla fruits are considered useful for increasing visual acuity during the night and easing circulation disorders [38]. With respect to the skin, the fruit is useful for treating mouth conditions, such as thrush and stomatitis [38]. Other authors described that murtilla fruits might have beneficial effects in the protection against cardiovascular diseases [39]. The authors described murtilla fruit extract as rich in quercetin-3-*β*-D-glucoside, gallic acid, myricetin, catechin, kaempferol, and quercetin, and they also showed that these extracts contain significant antioxidant activity against ROS production and lipid peroxidation, with no toxic effects on human endothelial cells (Table 3).

### 4.1. Anti-Inflammatory Properties of the Murtilla

The inflammation process is a response to metabolic syndrome, autoimmune diseases, neurodegenerative diseases, and cardiovascular diseases [40]. Chronic inflammation produces higher reactive oxygen species (ROS) accumulation that affects various proteins triggering the release of inflammatory signals [4, 41–43]. Different authors showed that natural products, such as PCs [42] and/or terpenoids [44, 45], possess anti-inflammatory effects.

**Figure 3:**
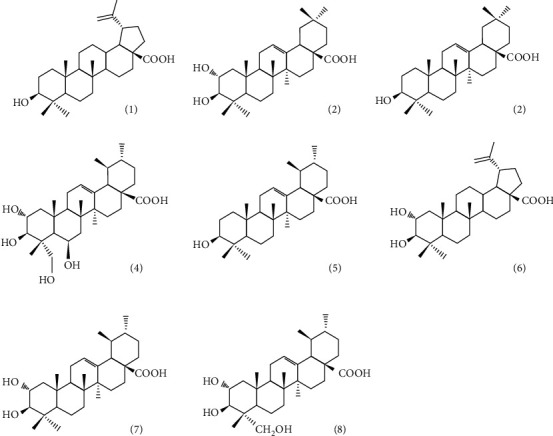
Structure of the principal triterpenes described from murtilla leaves: (1) betulinic acid, (2) maslinic acid, (3) oleanolic acid, (4) madecassic acid, (5) ursolic acid, (6) alphitolic acid, (7) corosolic acid, and (8) asiatic acid. The number of each structure is the same number given to the corresponding anthocyanin molecule described in Table 5.

Firstly, with respect to the terpenoid compounds, Goity et al. in 2013 [44] identified 2 triterpenoids (molecules 2 and 4 in Table 5 and Figure 3) from murtilla leaf extracts in ethyl acetate and ethanol. The authors observed that these triterpenoids are responsible in part for the anti-inflammatory activity in an assay on mice ear edema induced by 12-O-tetradecanoylphorbol-13-acetate (TPA) [44].

Then, in 2016, Arancibia-Radich et al. [45] studied the activity of two extracts from murtilla leaves with ethyl acetate or ethanol, identifying eight triterpenoids from ten different *Ugni molinae* genotypes (molecules 1 to 8 in Table 5 and Figure 3). The results obtained showed that there is a quantitative difference between triterpenoid content and total phenolic compounds from two extracts, showing significant differences in anti-inflammatory activity, due to the high phenolic content present in the methanolic extract [45]. Previously, in 2006, Aguirre et al. [7] isolated and identified three triterpene acids (molecules 6 to 8 in Table 5 and Figure 3). The authors also reported the activity of 2*α*-hydroxy pentacyclic triterpene acids from murtilla leaves. Initially, only corosolic acid (molecule 7 in Table 5 and Figure 3) showed an activity in the anti-inflammatory assay when an edema was induced by TPA in mice ears, in which the activity showed a similar level that was described by an anti-inflammatory COX-2 selectively named nimesulide [7]. Additionally, molecules 6 and 8 (from Table 5 and Figure 3) showed an inhibition of the TPA-induced inflammation comparable to indomethacin (a methylated indole derivative nonsteroidal anti-inflammatory drug related to diclofenac, which inhibits prostaglandin production) [7].

With respect to the phenolic compounds, different authors showed a high content of quercetin (a flavonoid compound) suggesting its protective effect against inflammatory diseases (Table 3). Quercetin is mainly found as quercetin 3-rutinoside in fruits and vegetables, and this compound was described in fruits such as murtilla and in other berries such as calafate, commercial strawberry, Chilean strawberry, raspberry, and blueberry [4, 16, 33].

### 4.2. Antibacterial Properties of the Murtilla Extract

Today, one of the main problems in the pharmaceutical industry is the increasing resistance of microorganisms, leading to treatment failures [45]. Thus, the discovery of new and better antimicrobial compounds is crucial, and the South American natives' berries, such as the murtilla fruits or leaves, are a good source of bioactive compounds. In this sense, extracts obtained from murtilla leaves and fruits have been used for different purposes. Junqueira-Gonçalves et al. in 2015 [46] determined the antimicrobial activity in extracts (obtained from ethanolic and methanolic extraction) from murtilla fruit over *E. coli* and *S. typhi* bacteria, showing that the extracts, obtained from methanol extraction, have an inhibitory activity against these bacteria very similar to that of standard antibiotics (Table 6) [46].

Additionally, extracts from murtilla leaves caused a decrease in the growth of *Pseudomonas aeruginosa*, *Klebsiella pneumoniae*, and *Staphylococcus aureus* (Table 7), and a significant correlation between PCs and antimicrobial activity over these bacteria was reported by Shene et al. in 2009 [47]. The authors showed a higher antimicrobial activity in leaf extracts than in fruit extracts. This could be attributed to the higher concentration of flavan-3-ols and other flavonol glycosides found in the leaves compared to that in the fruit [47].

## 5. New Perspectives and Challenges

Berries in general are among the most popular fruits consumed worldwide since they are available throughout the year as fresh and/or frozen products. For this reason, the berry production is now in continuous growth and improvement due to the increasing demand by global consumers who require healthier and functional food. Thus, the promotion of scientific research to innovate and improve berry production is necessary, especially in topics that strengthen the quality of fruit. Thus, murtilla berries emerge as a rich source of phytochemicals and vitamins and has been highly ranked among the dietary sources of polyphenols with antioxidant and antibacterial capacity.

An important point is the lack of genetic information about genome information and also a lack of information at the transcriptional level of the key genes related to the polyphenols or any other bioactive compound biosynthesis in murtilla. Thus, the lack of genetic information regarding the molecular mechanism commanding the bioactive compound formation during the development of the murtilla fruit highlights the importance of developing investigative frameworks to better understand their regulation. Progress in this arena can be useful in breeding programs that are necessary to improve the fruit quality and beneficial properties present in the native murtilla plants. Finally, with the currently available information about the murtilla fruit, it is possible to suggest that this fruit meets the requirements to reach the category of a new functional food and a good source of bioactive compounds.

## Figures and Tables

**Table 1:** Aroma volatile compound concentrations (*μ*g kg^−1^ fresh weight) from four ecotypes of the murtilla fruit at the initial storage temperature of 0°C. Table was adapted from Scheuermann et al. in 2008 [13], and the values represent mean ± standard deviation of the two replicates.

Volatile compound	Ecotype 14-4	Ecotype 17-2	Ecotype 19-1	Ecotype 33-5
Methyl 2-methyl butanoate	123.2 ± 2.1	127.1 ± 25.7	73.3 ± 0.4	80.5 ± 8.8
Ethyl butanoate	20.8 ± 0.5	161.6 ± 60.0	143.2 ± 0.4	41.3 ± 4.8
Methyl pentanoate	19.9 ± 1.0	12.3 ± 0.8	6.9 ± 0.3	9.7 ± 0.8
Ethyl 2-methyl butanoate	4.2 ± 0.5	80.0 ± 29.2	50.0 ± 3.5	11.7 ± 1.9
Methyl hexanoate	223.5 ± 33.2	250.5 ± 20.3	97.2 ± 8.9	147.5 ± 26.2
*α*-Pinene	4.6 ± 1.1	43.3 ± 53.1	55.9 ± 71.9	3.6 ± 0.2
Ethyl hexanoate	7.2 ± 1.4	99.8 ± 12.6	72.3 ± 6.5	18.9 ± 1.6
1,8-Cineole	7.0 ± 1.1	9.5 ± 0.3	7.1 ± 0.3	7.5 ± 0.6
D-Limonene	2.6 ± 0.4	72.7 ± 97.6	41.0 ± 53.5	3.5 ± 0.5
Methyl benzoate	87.6 ± 17.4	5.5 ± 0.3	158.5 ± 13.6	28.4 ± 1.2

**Table 2:** Proximal and chemical analyses of the *Ugni molinae* fruit.

Analysis type	Nutrient type	Per 100 g	References
Proximal	Water (%)	76.95–84.14	[48–50]
	Calories (kcal)	75	[25]
	Soluble solids (°Bx)	6.5–28	[13, 48, 49]
	pH	3.5–5.2	[13, 48, 49]
	Total protein (g)	1.15–17.98	[48–50]
	Ash content (g)	0.60–0.89	[48]
	Total lipid (g)	0.30–0.85	[48, 49]
	Crude fiber (g)	2.50–3.24	[48]
	Carbohydrate (g)	17.6–19.4	[48]
	Dietary fiber (g)	21.6	[22]
	Pectins (g)	0.32–1.14	[11]
Sugars	Total sugars (g)	58.2	[11]
Minerals	Calcium (mg)	90	[25, 26]
	Phosphorum (mg)	20	
	Potassium (mg)	116	
Vitamins	Vitamin C (mg)	4.53–2.55	[24]

**Table 3:** Principal phenolic compounds found in murtilla.

Plant source	Compound	Technique	References
*Flavonoids*			
Fruit	Quercetin	HPLC/ESI-MS and LC-PDA–HR-ToF-ESI-MS	[46, 51]
Leaves	Quercetin (ethanol extract)	HPLC-UV-VIS/MS, HPLC-UV-VIS, and HPLC/ESI-MS/MS	[52–54]
Fruit	Kaempferol	HPLC/ESI-MS	[46]
Leaves	Kaempferol (ethanol extract)	HPLC-UV-VIS/MS and HPLC-UV-VIS	[52–54]
Fruit	Isoquercitrin	HPLC-PDA–HR-ToF-ESI-MS	[51]
Fruit	Rutin	HPLC/ESI-MS and LC-PDA–HR-ToF-ESI-MS	[46, 51–55]
Leaves	Rutin (ethanol extract)	HPLC-UV-VIS/MS	[53]
Leaves	Rutin (ethanol 50% and water extract)	HPLC-PDA-FL	[55]
Leaves	Rutin (methanol extract)	HPLC ESI-MS/MS	[52]
Fruit	Myricetin	HPLC/ESI-MS and LC-PDA–HR-ToF-ESI-MS	[46, 51]
Leaves	Myricetin (methanol extract)	HPLC-UV-VIS/MS and HPLC ESI-MS/MS	[53, 56]
Leaves	Myricetin (ethanol extract)	HPLC-UV-VIS/MS	[54]
Fruit	Luteolin	HPLC/ESI-MS	[46]
Leaves	Epicatechin (methanol and ethanol extract)	HPLC-UV-VIS/MS	[56]
Leaves	Catechin (ethanol 50% extract)	HPLC-PDA-FL	[55]
Leaves	Catechin (infusion)	HPLC-UV-VIS	[8]
Leaves	Amentoflavone (ethanol extract)	HPLC-UV-VIS/MS	[54]

*Organic acids*			
Fruit	Gallic acid	HPLC/ESI-MS	[46]
Leaves	Gallic acid (infusion)	HPLC-UV-VIS	[8]
Leaves	Gallic acid (ethanol extract)	HPLC-UV-VIS/MS	[54]
Leaves	Ácido elágico (infusion)	HPLC-UV-VIS	[8]
Leaves	Ellagic acid pentoside (ethanol extract)	HPLC-UV-VIS/MS	[54]
Leaves	Chlorogenic acid (ethanol extract)	HPLC-UV-VIS/MS	[54]
Leaves	Digalloyl quinic acid (ethanol extract)	HPLC-UV-VIS/MS	[54]

*Cinnamic acids and derivatives*			
Fruit	*p*-Coumaric acid	HPLC/ESI-MS	[46]
Fruit	Caffeic acid 3-glucoside	HPLC/ESI-MS	[46]
Fruit	Feruloyl-quinic acid	HPLC-PDA–HR-ToF-ESI-MS	[51]
Fruit	Chlorogenic acid	HPLC-PDA–HR-ToF-ESI-MS	[51]
Leaves	Sinapic acid hexoside (ethanol extract)	HPLC-UV-VIS/MS	[54]

*Flavonoid glycosides*			
Fruit	Cyanidin-3-glucoside	HPLC/ESI-MS	[46]
Fruit	Pelargonidin-3-arabinose	HPLC/ESI-MS	[46]
Fruit	Delphinidin-3-glucoside	HPLC/ESI-MS	[46]
Fruit	Quercetin-3-glucoside	HPLC/ESI-MS	[46]
Leaves	Quercetin-3-glucoside (water, methanol, and ethanol extract)	HPLC-UV-VIS/MS	[56]
Fruit	Kaempferol-3-glucoside	HPLC/ESI-MS	[46]
Leaves	Kaempferol-3-glucoside (water, methanol, and ethanol extract)	HPLC-UV-VIS/MS	[56]
Fruit	Luteolin-3-glucoside	HPLC/ESI-MS	[46]
Leaves	Myricetin dirhamnoside (water, methanol, and ethanol extract)	HPLC-UV-VIS/MS	[56]
Leaves	Myricetin glucoside (water, methanol, and ethanol extract)	HPLC-UV-VIS/MS	[56]
Leaves	Quercetin dirhamnoside (methanol and ethanol extract)	HPLC-UV-VIS/MS	[56]
Leaves	Quercetin rhamnoside (methanol and ethanol extract)	HPLC-UV-VIS/MS	[56]
Leaves	Myricetin rhamnoside (water, methanol, and ethanol extract)	HPLC-UV-VIS/MS	[56]
Leaves	Myricetin rhamnoside (ethanol extract)	HPLC-UV-VIS/MS	[54]
Leaves	Myricetin xyloside (water extract)		
Fruit	Neochlorogenic acid	LC-PDA–HR-ToF-ESI-MS	[53]
Fruit	Hyperoside	LC-PDA–HR-ToF-ESI-MS	[53]
Leaves	Galloyl myricetin hexoside (ethanol extract)	HPLC-UV-VIS/MS	[54]
Leaves	Quercitrin (ethanol 50% and water extract)	HPLC-PDA-FL	[55]

*Isoflavones*			
Leaves	Daidzin (methanol extract)	HPLC/ESI-MS/MS	[52]
Leaves	Formononetin (methanol extract)	HPLC/ESI-MS/MS	[52]
Leaves	Biochanin A (methanol extract)	HPLC/ESI-MS/MS	[52]
Leaves	Genistein (methanol extract)	HPLC/ESI-MS/MS	[52]

HPLC: high-performance liquid chromatography; ESI-MS: electrospray ionization mass spectrometry; UV-VIS: ultraviolet-visible spectroscopy; PDA: photodiode detector; ToF/MS: time of flight mass spectrometry; HR-MS: high-resolution mass spectrometry.

**Table 4:** Principal anthocyanin compound found in murtilla fruit.

No.	Plant source	Compound	Technique	References
1	Fruit	Petunidin 3-O-galactoside	LC/MS, LC-PDA–HR-ToF-ESI-MS, and HPLC-PDA	[34, 46, 51]
2	Fruit	Delphinidin 3-O-galactoside	LC/MS	[46]
3	Fruit	Delphinidin 3-O-arabinoside	LC/MS	[46]
4	Fruit	Cyanidin 3-O-glucoside	LC/MS and LC/MS ESI-IT	[46, 57]
5	Fruit	Cyanidin 3-O-arabinoside	LC/MS	[47]
6	Fruit	Malvidin 3-O-arabinoside	LC/MS	[47]
7	Fruit	Peonidin-malvidin 3-O-glucoside	LC/MS	[47]
8	Fruit	Peonidin 3-O-glucoside	LC/MS, LC-PDA–HR-ToF-ESI-MS, and HPLC-PDA	[34, 46, 51]
9	Fruit	Cyanidin 3-O-rutinose	LC-PDA–HR-ToF-ESI-MS and HPLC-PDA	[34, 51]
10	Fruit	Petunidin-3-O-rutinoside	LC-PDA–HR-ToF-ESI-MS and HPLC-PDA	[34, 51]
11	Fruit	Peonidin-3-O-arabinoside	LC-PDA–HR-ToF-ESI-MS and HPLC-PDA	[34, 51]
12	Fruit	Cyanidin-3-O-(6^″^ succinoyl)-glucose	LC-PDA–HR-ToF-ESI-MS and HPLC-PDA	[34, 51]
13	Fruit	Peonidin-di-glucopyranoside	LC/MS ESI-IT	[57]
14	Fruit	Peonidin glucopyranoside	HPLC-PDA	[34]

HPLC: high-performance liquid chromatography; ESI-MS: electrospray ionization mass spectrometry; UV-VIS: ultraviolet-visible spectroscopy; PDA: photodiode detector; ToF/MS: time of flight mass spectrometry; HR-MS: high-resolution mass spectrometry; ESI-IT: electrospray ionization- (ESI-) ion trap (IT).

**Table 5:** Principal triterpenoid compounds found in murtilla leaves.

No.	Plant source	Compound	References
1	Leaves	Betulinic acid	[45]
2	Leaves	Maslinic acid	[44, 45]
3	Leaves	Oleanolic acid	[45]
4	Leaves	Madecassic acid	[44, 45, 54]
5	Leaves	Ursolic acid	[45]
6	Leaves	Alphitolic acid	[7, 45]
7	Leaves	Corosolic acid	[7, 45]
8	Leaves	Asiatic acid	[44, 45, 54]

**Table 6:** Summary of antibiotics against *S. typhi* and *E. coli*. The activity was registered in inhibition halo diameter (mm) and Mueller-Hinton's agar medium. *n* = mean of three independent experiments (adapted from Junqueira-Gonçalves et al. in 2015 [46]).

Plant source	Type extract	Concentration	Antibiotics	*E. coli*	*S. typhi*
	Commercial product	Not informed	Tetracycline	28.3 ± 0.9	23.7 ± 0.4
		Not informed	Gentamicin	10.7 ± 0.3	24.3 ± 0.3
		Not informed	Ampicillin	22.3 ± 0.9	22.7 ± 0.3
Fruit	Methanolic :water :HCl (75 : 24 : 1)	100 *μ*L		No reported value	22.5

**Table 7:** Summary of *S. aureus* ATCC 25923, *K. pneumoniae* ATCC 13883, and *P. aeruginosa* ATCC 27853. The activity was registered in inhibition halo diameter (mm) and Mueller-Hinton's agar medium. *n* = mean of three independent experiments (adapted from Shene et al. in 2009 [47]).

Plant source	Type extract	Concentration	Antibiotics	*S. aureus* ATCC 25923	*K. pneumoniae* ATCC 13883	*P. aeruginosa* ATCC 27853
Fruit	Ethanol	100 *μ*L	—	1.1 ± 0.3^a^	—	—
				0.9 ± 0.3^b^		
				1.0 ± 0.3^c^		
Leave		100 *μ*L	—	39.7 ± 8.6^a^	18.5 ± 1.1^a^	55.8 ± 10.9^a^
				26.6 ± 4.7^b^	17.8 ± 7.4^b^	53.4 ± 6.7^b^
				43.7 ± 16.3^c^	22.2 ± 4.4^c^	81.1 ± 3.8^c^

Letters indicate the location where the murtilla plants were obtained: (a) mountain, (b) coast, and (c) valley.

## Data Availability

The requirement for data availability does not apply, because this is a review.
